# [Retracted] Growth and differentiation factor-9 promotes adhesive and motile capacity of prostate cancer cells by up-regulating FAK and Paxillin via Smad dependent pathway 

**DOI:** 10.3892/or.2026.9089

**Published:** 2026-03-02

**Authors:** Sivan M. Bokobza, Lin Ye, Howard G. Kynaston, Wen G. Jiang

Oncol Rep 24: 1653–1659, 2010; DOI: 10.3892/or_00001030

Subsequently to the publication of the above article, a concerned reader drew the Editor's attention to the fact that certain of the protein bands shown in lanes 1, 3 and 4 for the Smad3 experiments for the western blots in Fig. 5A on p. 1657 were strikingly similar; furthermore, the protein bands shown in lanes 2 and 5 also appeared to be highly similar aside from a difference in the intensity of the shading (in conjunction with probable breaks in the continuity of the gel). In addition, the backgrounds of lanes 3 and 4 for the pSmad3 data shown in Fig. 5C were markedly similar in appearance.

Having assessed these data independently in the Editorial Office, the concerns of the reader were judged to have been well founded, and the striking similarities in the appearance of the abovementioned protein bands/gel backgrounds could not easily be attributed to coincidence. Therefore, in view of the probable anomalies that were identified, the Editor of *Oncology Reports* has decided that this paper should be retracted from the Journal on account of a lack of confidence in the data. The authors were asked for an explanation to account for these concerns, but the Editorial Office did not receive a reply. The Editor apologizes for any inconvenience caused.

